# Contemporary Perspectives on the Role of Vitamin D in Enhancing Gut Health and Its Implications for Preventing and Managing Intestinal Diseases

**DOI:** 10.3390/nu16142352

**Published:** 2024-07-20

**Authors:** Jiaxin Wang, Lihua Mei, Yanling Hao, Yajun Xu, Qing Yang, Zhaolai Dai, Ying Yang, Zhenlong Wu, Yun Ji

**Affiliations:** 1 State Key Laboratory of Animal Nutrition and Feeding, China Agricultural University, Beijing 100193, China; wangjiaxin202309@163.com (J.W.); meilh329@163.com (L.M.); qing.yang@cau.edu.cn (Q.Y.); daizhaolai@cau.edu.cn (Z.D.); cauvet2020@hotmail.com (Y.Y.); wuzhenlong@cau.edu.cn (Z.W.); 2 Beijing Advanced Innovation Center for Food Nutrition and Human Health, China Agricultural University, Beijing 100193, China; haoyl@cau.edu.cn; 3 Department of Nutrition and Food Hygiene, Peking University, Beijing 100083, China; xuyajun@bjmu.edu.cn

**Keywords:** vitamin D, gut microbiome, intestinal barrier, IBD, IBS

## Abstract

Vitamin D, a crucial fat-soluble vitamin, is primarily synthesized in the skin upon exposure to ultraviolet radiation and is widely recognized as a bone-associated hormone. However, recent scientific advancements have unveiled its intricate association with gut health. The intestinal barrier serves as a vital component, safeguarding the intestinal milieu and maintaining overall homeostasis. Deficiencies in vitamin D have been implicated in altering the gut microbiome composition, compromising the integrity of the intestinal mucosal barrier, and predisposing individuals to various intestinal pathologies. Vitamin D exerts its regulatory function by binding to vitamin D receptors (VDR) present in immune cells, thereby modulating the production of pro-inflammatory cytokines and influencing the intestinal barrier function. Notably, numerous studies have reported lower serum vitamin D levels among patients suffering from intestinal diseases, including inflammatory bowel disease, irritable bowel syndrome, and celiac disease, highlighting the growing significance of vitamin D in gut health maintenance. This comprehensive review delves into the latest advancements in understanding the mechanistic role of vitamin D in modulating the gut microbiome and intestinal barrier function, emphasizing its pivotal role in immune regulation. Furthermore, we consolidate and present relevant findings pertaining to the therapeutic potential of vitamin D in the management of intestinal diseases.

## 1. Introduction

Vitamin D, a fat-soluble vitamin and secosteroid hormone, is a vital nutrient that plays a crucial role in various physiological processes within the human body. It is synthesized in the skin upon exposure to ultraviolet B (UV-B) radiation from sunlight and can also be obtained through the diet, primarily from fatty fish, fortified dairy products, and supplements [1]. Beyond its well-established functions in regulating calcium and phosphorus metabolism and its importance in bone health, vitamin D has emerged as a key player in a multitude of physiological and pathological conditions.

Recent studies have provided insights into the complex connection between vitamin D and the intestinal tract, which is the largest and most intricate immunological and digestive organ in the body [2]. The gut microbiota, a complex and ever-changing community of microorganisms, resides in the intestinal tract. It plays a crucial role in breaking down nutrients, producing vitamins, and maintaining the immune balance of the gut mucosa [3]. The gut microbiota and the host’s immune system maintain a delicate equilibrium, and any disruption can potentially trigger a series of intestinal diseases, such as inflammatory bowel diseases, irritable bowel syndrome, etc. [4,5]. Emerging findings indicate that vitamin D plays a substantial role in shaping the composition of the gut microbiota and maintaining the integrity of the intestinal mucosal barrier. The existence of vitamin D receptors (VDR) throughout the intestinal tract highlights the significance of this nutrient in preserving gut equilibrium. Acting as a transcription factor, VDR governs the expression of genes associated with the biological effects of vitamin D [6]. Vitamin D exerts regulatory control over the microbiome in both health and disease states via vitamin D and VDR signal transduction pathways, crucial for maintaining gut health [7]. Vitamin D has been demonstrated to regulate the gut microbiota by stimulating the growth of advantageous bacteria and suppressing the proliferation of potentially detrimental species [8]. This modulation can augment the inherent protective function of the intestinal tract, thereby inhibiting the movement of harmful microorganisms and the subsequent triggering of the immune response. Moreover, vitamin D plays a role in regulating the immune response in the intestinal tract. It has the ability to impact both the adaptive and innate immune systems by controlling the functioning of different immune cells, such as T cells, B cells, and macrophages [9]. The immunomodulatory role is essential for maintaining a harmonized immune response and preventing excessive activation of the immune system, which can result in persistent inflammation and tissue harm in the intestinal tract.

Inflammatory bowel disease (IBD) individuals, particularly those with ulcerative colitis (UC), exhibit low serum vitamin D concentrations (25-hydroxyvitamin D_3_ [25(OH)D_3_] ≤ 20 ng/mL), according to a meta-analysis and systematic review [10]. Ensuring sufficient levels of vitamin D can be advantageous not only in preventing these conditions but also in managing existing intestinal diseases. The potential therapeutic efficacy of vitamin D in promoting intestinal health is a subject of increasing interest. For example, multiple studies indicate that vitamin D supplementation may enhance symptoms and decrease the recurrence rate in individuals with inflammatory bowel disease [11,12].

Comprehending the intricate relationship between vitamin D and the gut is crucial for devising innovative therapeutic approaches to enhance gut health and avert intestinal diseases. This review aims to provide an overview of the current understanding of the role of vitamin D in gut health and disease. We will discuss the mechanisms by which vitamin D influences the gut microbiota, intestinal barrier function, and immune response, and explore the clinical implications of vitamin D deficiency and supplementation in the context of intestinal diseases.

## 2. Understanding Vitamin D: Definition and Sources

**Figure 1:**
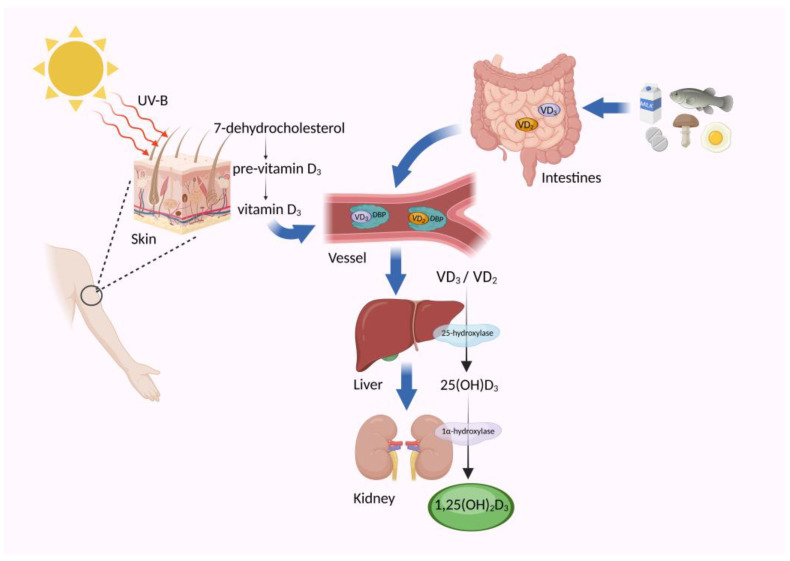
The synthesis, sources, and metabolism of vitamin D. Under the influence of UV-B radiation, the 7-dehydrocholesterol in the skin undergoes conversion into pre-vitamin D, subsequently transforming into vitamin D_3_ through body temperature-mediated processes. Alternatively, dietary supplements can also serve as sources of vitamin D. Notably, vitamin D_2_ is sourced from plants, whereas vitamin D_3_ originates from animals. Upon entering the bloodstream, vitamin D_2_/D_3_ binds to the vitamin D binding protein (DBP), facilitating its transportation to the liver and kidneys. Within these organs, it undergoes successive transformations, ultimately culminating in the formation of biologically active 1,25-dihydroxyvitamin D_3_ (1,25(OH)D_3_). This active form is then circulated throughout the body via the bloodstream, delivering its vital functions to various organs and tissues. Created with Biorender.com (accessed on 18 July 2024).

Vitamin D, a steroid and fat-soluble vitamin essential for human health, plays a crucial role in various physiological processes. Its primary synthesis occurs through sunlight exposure on the skin and dietary intake. When the skin is exposed to ultraviolet radiation, 7-dehydrocholesterol is converted into pre-vitamin D_3_, subsequently transformed into vitamin D_3_ with the aid of body temperature [1]. This synthesized vitamin D_3_ enters the bloodstream, binding with the vitamin D binding protein (DBP), and then is transported to the liver. Within the liver, it undergoes conversion by microsomal 25-hydroxylase into 25-hydroxyvitamin D_3_ [25(OH)D_3_]. This form of vitamin D, still bound to the DBP, is further transported to the renal tubular epithelial cells in the kidney [13]. There, mitochondrial 1α-hydroxylase catalyzes its conversion into the biologically active form, 1,25-dihydroxyvitamin D_3_ [1,25(OH)_2_D_3_], which predominates in the body. 1,25(OH)_2_D_3_ circulates throughout the body via the bloodstream, reaching various tissues and organs [14,15,16]. In instances where endogenous synthesis of vitamin D is inadequate, dietary supplementation becomes necessary to maintain optimal levels. Vitamin D obtained from dietary sources accompanies fat into the digestive tract and is absorbed predominantly in the jejunum and ileum, facilitated by bile action. Once absorbed, vitamin D, along with chylomicrons, enters the bloodstream via the lymphatic system or binds to specific vitamin D transporters. Eventually, it undergoes activation primarily in the liver and kidneys before entering systemic circulation [17] (Figure 1).

Vitamin D is integral to the regulation of calcium and phosphorus metabolism, crucial for promoting bone resorption, and pivotal in facilitating body growth and bone development. Beyond its fundamental skeletal functions, an extensive body of research underscores its intricate involvement in various physiological processes [18]. Numerous studies have elucidated the intricate relationship between vitamin D and a spectrum of health conditions, including obesity, autism, respiratory diseases, digestive tract disorders, cardiovascular ailments, cancer, and multiple sclerosis [19,20,21,22,23]. Furthermore, recent investigations have unveiled additional roles of vitamin D in modulating cellular proliferation and differentiation [24], orchestrating hormone secretion [25], shaping the structure of the gut microbiome [26], fortifying intestinal barrier function [27], and optimizing immune system functionality [28,29]. Thus, the multifaceted influence of vitamin D extends beyond its classical roles, highlighting its indispensable significance in maintaining overall health and well-being.

## 3. The Dynamic Interplay: Impact of Vitamin D on the Gut Microbiome and Intestinal Barrier Function

The intestinal tract, comprising the largest digestive organ in the human body, serves as a critical site for food digestion and nutrient absorption. In conjunction with endogenous digestive secretions, the gut harbors a vast array of microorganisms, pivotal in fostering gut and overall bodily health. A thriving gut ecosystem is instrumental in the efficient breakdown and assimilation of nutrients essential for growth and maintenance. Moreover, the gut boasts extensive immunological activity, adept at discerning and combatting pathogens. Sustaining gut homeostasis bolsters immune equilibrium, averting hyperimmunity and chronic inflammation. Conversely, dysbiosis within the gut microbiome and disruption of the intestinal mucosal barrier may precipitate immune hyperactivity, culminating in inflammatory and autoimmune pathologies [30,31]. In addition, emerging research underscores the intricate interplay between the gut microbiome and various physiological processes, including lipid metabolism and communication with the central nervous system via the microbiota–gut–brain axis, thereby implicating its profound implications for mental health and neurological disorders [32,33]. Optimal levels of vitamin D are essential for maintaining gut health, influencing the composition of the gut microbiome and the integrity of the intestinal mucosal barrier. The multifaceted impact of vitamin D encompasses enhancing the stability and diversity of the gut microbiome, reinforcing the functionality of the intestinal mucosal barrier, modulating immune cell activity, and preserving mucosal integrity [34,35]. Several recent studies have identified gut-microbiota-dysbiosis-associated metabolic disorders as possible triggers or causes for dermatological conditions [36,37]. These findings suggest a broader role of vitamin D in modulating tissue functions via the gut microbiome.

### 3.1. Regulation of Vitamin D on the Composition of the Gut Microbiota

The gut harbors a diverse and intricate microbial ecosystem known as the gut microbiome, which is inherently host-specific. Its composition and species vary across different microenvironments and species. Through continuous selection and coadaptation during the interaction between the host and its environment, the gut microbiome gradually attains dynamic stability, playing a pivotal role in host body adaptation and equilibrium. This delicate balance can be perturbed by various factors such as genetics, age, antibiotics, and environmental influences [7].

The interactions between vitamin D and the gut microbiome are complex, and studies have demonstrated the capacity of vitamin D to modulate and improve the gut microbiome under both diseased and healthy conditions [38]. For instance, a non-randomized trial supplementing 80 vitamin D-deficient women with 50,000 IU of vitamin D per week for 12 weeks significantly augmented the richness and diversity of their gut microbiomes [39]. In the obese population, a Mediterranean lifestyle intervention was able to increase serum vitamin D levels, which is associated with increased gut microbiota diversity, as well as specific gut microbiota profiles [40]. Conversely, deficiency in VDR not only precipitates metabolic disorders in mice but also alters the structure of the gut microbiome and virome, influencing virus–microbiome interactions [41,42]. Yet, conflicting evidence exists; while some studies indicate that long-term vitamin D supplementation (60,000 IU/month) does not significantly alter gut microbiome composition and diversity [43], others suggest potential effects, albeit at a lower taxonomic level [44]. It may be that factors such as the age and gender of the participants have contributed to the inconsistent effects of vitamin D and gut microbiome interactions [45,46]. Hence, the extent to which vitamin D supplementation impacts the composition of the gut microbiome remains a subject of ongoing investigation. The regulation of the gut microbiome by vitamin D is further supported by the additional evidence provided in Table 1.

### 3.2. Contribution of the Gut Microbiome to Vitamin D Metabolism

**Figure 2:**
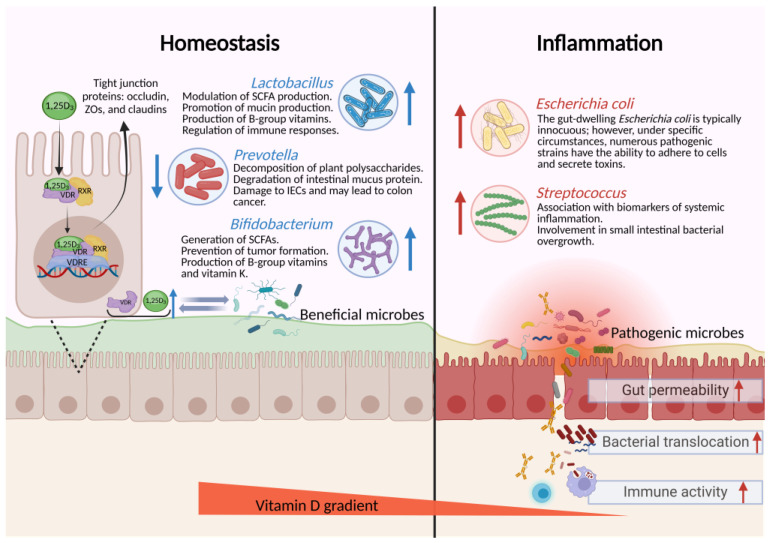
Unlocking the nexus: vitamin D, gut microbes, and epithelial cell dynamics. The vitamin D receptors (VDR) are highly abundant in intestinal epithelial cells [58]. Following the entry of vitamin D into intestinal epithelial cells, VDR can form a heterodimer with RXR. This complex translocates to the nucleus and binds to the vitamin D response element (VDRE) in the promoter region of the target genes, thereby regulating VDR expression. Interactions between vitamin D/VDR and intestinal microbes are observed. On one hand, they enhance tight junction protein expression, maintain intestinal epithelial tissue integrity, reduce bacterial translocation, promote colonization by beneficial bacteria, and alleviate mucosal damage and abnormal immune activation [59,60,61]. On the other hand, the gut microbiota can produce lithocholic acid (LCA), which facilitates vitamin D absorption. Created with Biorender.com (accessed on 18 July 2024).

Research has shown a correlation between serum 1,25(OH)_2_D_3_ levels and the shifts in bacterial community composition or gut microbiota diversity, indicating a role of the microbiome in regulating vitamin D metabolism and circulation in the gut [52]. While the gut microbiome does not possess the VDR, the gut microbiota is associated with the expression of VDR, which in turn affects circulating vitamin D levels. For instance, in both murine and human intestinal epithelial cells, supplementation with *Lactiplantibacillus plantarum* and *Limosilactobacillus reuteri* increased VDR protein expression, augmented Paneth cell numbers, and bolstered mice resilience against salmonella-induced colitis in mice [53]. Moreover, human studies have shown that supplementation with *Lactobacillus reuteri* for a duration of 9 weeks led to elevated serum 25(OH) vitamin D_3_ levels [54]. In obese patients, serum vitamin D levels were significantly positively correlated with Bifidobacteria abundance in the gut [55]. Conversely, an increase in gut pathogens has been associated with the down-regulation of VDR expression [56] (Figure 2). In addition, the gut microbiota can produce lithocholic acid (LCA), which facilitates intestinal absorption of vitamin D, consequently enhancing its bioavailability [57].

### 3.3. Role of Vitamin D in Preserving Intestinal Barrier Function

The intestinal barrier constitutes a multifaceted structure comprising the gut microbiome, mucus layer, epithelial cell layer, and immune cell layer, playing a pivotal role in both health and disease. Any impairment in barrier function or subtle alterations in microbial, mucus, epithelial, or immune components may contribute to the pathogenesis of complex diseases [62]. As the largest immune organ in the body, the gut harbors various specialized structures such as plicae circulares, intestinal villi, and crypts within the intestinal mucosa, where the mucus layer, epithelial cells, immune cells in the lamina propria, and tight junctions work in concert. The intimate association between the intestinal mucosa and the gut microbiome fosters a symbiotic relationship crucial for immune homeostasis [63,64]. The gut microbiome exerts protective effects by competitively inhibiting the attachment and proliferation of pathogenic bacteria, while also aiding in nutrient breakdown and synthesis of essential vitamins and amino acids necessary for host health [65,66]. The mucus layer, enriched with mucin, antimicrobial peptides, and secretory immunoglobulin A (sIg A), serves as the initial physical barrier encountered by luminal contents, thereby impeding direct contact between bacteria and epithelial cells and mitigating intestinal inflammation [67].

Intestinal epithelial cells play a pivotal role in maintaining the integrity of the barrier by establishing a selectively permeable interface between the luminal environment and the internal milieu. Apart from facilitating nutrient absorption and electrolyte balance, they serve as a formidable barrier against harmful substances while preserving immunological vigilance against potentially harmful compounds [67,68]. Moreover, the epithelial cell layer exhibits specialized immune and endocrine functions, further contributing to barrier integrity and immune surveillance. The immune cell layer, situated within the lamina propria beneath the epithelial cells, comprises diverse immune cells, including macrophages, dendritic cells, T cells, and mast cells, among others, each endowed with specific secretory and immunomodulatory roles, collectively orchestrating immune responses within the gut microenvironment [69].

Vitamin D exerts profound regulatory effects on immune cell development and function. It facilitates the differentiation of mononuclear macrophages into dendritic cells (DCs) and antigen-presenting cells (APCs), thereby augmenting their capacity for antigen capture and presentation [70,71]. Moreover, vitamin D promotes the differentiation of T cells into regulatory T cells (Tregs) while inhibiting the differentiation of inflammatory Th1 and Th17 cells [72,73]. Tregs, through the secretion of inhibitory cytokines such as transforming growth factor-β (TGF-β) and interleukin-10 (IL-10), modulate immune responses, dampen inflammatory reactions, and mitigate autoimmune diseases [74]. Th1 and Th17 cells, on the other hand, are T helper cell subpopulations implicated in the activation of B cells, neutrophils, and macrophages, as well as the mediation of inflammatory responses by inducing various pro-inflammatory factors, chemokines, and cell adhesion molecules [75,76]. Dysregulation of Th1 and Th17 cells is associated with inflammatory and autoimmune diseases [77,78]. On the one hand, vitamin D can inhibit the activation of intestinal immune cells (such as T cells) and reduce the production of inflammatory mediators, thereby reducing the intensity and duration of the intestinal immune response [74]. Specifically, vitamin D inhibits the production of pro-inflammatory cytokines, including interleukin-6 (IL-6), interleukin-17 (IL-17), and tumor necrosis factor-α (TNF-α), thereby curbing inflammatory damage within the gut [79,80]. Furthermore, vitamin D promotes the synthesis of the anti-inflammatory cytokine interleukin-10 (IL-10), which exerts immunosuppressive effects by dampening pro-inflammatory mediator production in macrophages and T cells, thereby enhancing immune regulation [81].

**Figure 3:**
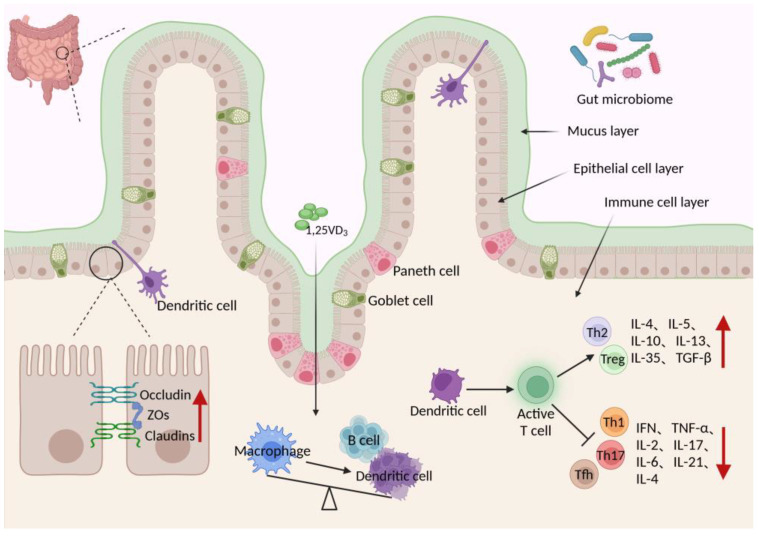
Modulation of intestinal barrier and mucosal immunity by vitamin D. Upon entering the intestine, vitamin D plays a crucial role in modulating immune responses by facilitating the differentiation of mononuclear macrophages into B cells and dendritic cells. This process significantly enhances the recognition and presentation of antigens. Furthermore, vitamin D promotes the transformation of dendritic cells into T cells, steering their differentiation towards Th2 and Treg phenotypes. This shift is accompanied by an increase in the secretion of anti-inflammatory cytokines such as IL-10 and TGF-β, while inhibiting the differentiation into pro-inflammatory Th1, Th17, and Tfh cells. Notably, vitamin D also downregulates the secretion of pro-inflammatory cytokines, including IFN, TNF-α, IL-17, and IL-6, further attenuating inflammatory responses. Additionally, it upregulates the expression of tight junction proteins such as occludin, zonula occludens (ZOs), and claudins, thereby reinforcing intestinal barrier integrity. Created with Biorender.com (accessed on 18 July 2024).

In addition to its immunoregulatory role, vitamin D enhances the integrity of the physical barrier within the intestinal mucosa. Tight junctions (TJs), composed of various proteins, including occludin, claudins, and zonula occludens (ZOs), constitute crucial adhesive structures between intestinal epithelial cells, imparting a vital physical barrier for mucosal immunity [82]. Disruption of TJ integrity leads to increased cellular permeability, exposure to bacterial toxins, and heightened pro-inflammatory cytokine release, culminating in immune cell activation and chronic inflammation [83,84]. Vitamin D preserves TJ expression in epithelial cells, promotes tight junction protein synthesis, prevents epithelial cell apoptosis, fortifies intercellular connections, and shields the intestinal mucosa from harmful microorganisms and toxins, thereby mitigating inflammation [35,85] (Figure 3).

### 3.4. Consequences of Vitamin D Deficiency on Intestinal Barrier Function

An increasing body of evidence suggests that vitamin D deficiency can disrupt the integrity of the intestinal mucosal barrier [58,86,87,88]. Studies conducted in mouse models of induced colitis have revealed significant alterations in intestinal barrier function among mice deficient in vitamin D compared to those with sufficient levels. Specifically, deficiencies in vitamin D are associated with heightened intestinal permeability, deeper crypt mucosal hyperplasia, and increased susceptibility to viral infections [27,89]. In vitro studies further support the protective role of vitamin D supplementation, demonstrating its ability to mitigate the escalation of paracellular permeability and redistribution of tight junctions in human colonic epithelial cells induced by pathogens such as *Escherichia coli*, thereby preserving the structural and functional homeostasis of the intestinal epithelium [90]. Moreover, vitamin D deficiency not only exacerbates disease activity but also contributes to low-grade inflammation. Research findings suggest that infection with *Clostridium* induces colonic epithelial cell proliferation and perturbs paracellular permeability in rodent models. However, compared to healthy counterparts with adequate vitamin D levels, uninfected mice lacking vitamin D exhibit heightened proliferative responses, increased permeability, and elevated expression of both pro-inflammatory and anti-inflammatory cytokines in colon tissue [87]. These findings underscore the critical role of vitamin D in maintaining intestinal barrier integrity and immune homeostasis, highlighting its potential as a therapeutic target for managing intestinal diseases.

## 4. Unraveling the Nexus: Association of Vitamin D with Gut Diseases

**Figure 4:**
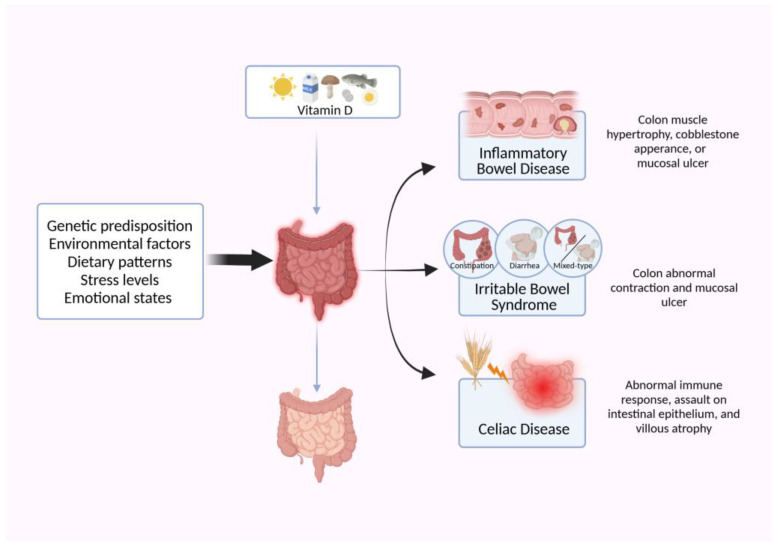
Factors affecting gut health and the role of vitamin D in gut diseases. Various factors including genetic predisposition, environmental influences, dietary patterns, stress levels, and emotional states contribute to the development of gut-related diseases. In this context, vitamin D supplementation has emerged as a promising intervention for alleviating conditions such as inflammatory bowel disease (IBD), irritable bowel syndrome (IBS), and celiac disease (CeD). IBD primarily manifests through colonic muscle hypertrophy, epithelial tissue fissures, pebble-like alterations (ulcerative colitis, UC), and mucosal injury (Crohn’s disease, CD). IBS patients often experience abnormal spasms in the large intestine and mucosal damage, leading to symptoms of constipation (IBS-C), diarrhea (IBS-D), or a combination of both (IBS-M). CeD, primarily affecting susceptible individuals consuming gluten, involves abnormal activation of intestinal mucosal immunity, consequently compromising the integrity of the intestinal barrier. Created with Biorender.com (accessed on 18 July 2024).

Vitamin D emerges as a pivotal factor in maintaining intestinal well-being and in the prevention and management of associated diseases. Apart from its recognized efficacy in ameliorating inflammatory bowel diseases (IBD), such as Crohn’s disease (CD) and ulcerative colitis (UC), vitamin D demonstrates promising utility in mitigating symptoms and complications of irritable bowel syndrome (IBS) and celiac disease (CeD) [91,92]. Notably, inadequate vitamin D levels are implicated in heightened susceptibility to a spectrum of intestinal ailments, encompassing IBD, IBS, and CeD (Figure 4). Interventions involving vitamin D supplementation exhibit therapeutic potential, engendering improvements in clinical manifestations and enhancing the quality of life for affected individuals [93,94,95]. Consequently, maintaining optimal vitamin D status assumes paramount importance in safeguarding intestinal health and in the prophylaxis and management of intestinal diseases.

### 4.1. Vitamin D and Inflammatory Bowel Disease (IBD)

IBD comprises a group of chronic, idiopathic intestinal inflammatory conditions, notably encompassing UC and CD [96]. UC manifests as mucosal inflammation extending from the rectum to the proximal colon, typified by hemorrhagic diarrhea [97]. By contrast, CD manifests as segmental, asymmetrical, and transmural inflammation, commonly affecting the ileum and colon and presenting with symptoms such as abdominal pain, chronic diarrhea, weight loss, and fatigue [98]. The etiology of IBD remains elusive, though it is believed to result from an aberrant immune response to intestinal antigens, influenced by a complex interplay of genetic predisposition, environmental factors, and gut microbiome dysbiosis. Notably, intestinal infection and immune dysregulation are deemed pivotal in disease pathogenesis [99,100]. Traditionally, IBD has exhibited a higher prevalence in North America, Northern Europe, and select Western nations compared to Asia, Southern Europe, and Africa. However, recent years have witnessed a rising incidence of IBD in Asian countries [101].

Beyond its classical roles in calcium and phosphorus metabolism, vitamin D assumes a critical function in modulating immune system dysregulation, rectifying gut microbiome imbalances, and preserving the integrity of the intestinal mucosal barrier [102,103,104]. Given its broad-spectrum antimicrobial and immunomodulatory properties, the role of vitamin D in IBD pathophysiology has garnered increasing attention and elucidation.

The biological actions of vitamin D are predominantly mediated via the VDR, which governs mucosal barrier homeostasis by upholding the integrity of junctional complexes and promoting colonic epithelial healing. Consequently, vitamin D exerts regulatory influence over intestinal barrier integrity, mucosal immunity, and gut microbiome composition [105]. Vitamin D deficiency may compromise mucosal barrier function, heightening susceptibility to mucosal injury and predisposing to IBD [88]. Indeed, studies have documented a high prevalence of vitamin D deficiency in the IBD population, with murine models of vitamin D deficiency demonstrating impaired antibacterial activity and heightened susceptibility to chemically induced colitis [106]. A comprehensive systematic review encompassing 27 studies and 8316 IBD patients corroborated the association between low vitamin D levels and disease activity, mucosal inflammation, quality of life scores, and prognostic outcomes in adult IBD patients [107].

As per the American Institute of Medicine (IOM) guidelines, vitamin D deficiency is defined by serum 25 (OH)D_3_ levels below 20 ng/mL (50 nmol/L) [108]. Notably, individuals with IBD often struggle to attain adequate vitamin D levels through dietary sources or sunlight exposure, necessitating oral supplementation [109]. In pediatric IBD patients, vitamin D supplementation has demonstrated significant efficacy in lowering serum C-reactive protein (CRP) levels and erythrocyte sedimentation rates (ESR), concurrently elevating serum 25 (OH)D_3_ and calcium levels. Moreover, higher supplemental doses exceeding 2000 IU per week have shown superior therapeutic outcomes [110]. Similarly, in adult IBD patients, supplementation with 40,000 IU of vitamin D weekly for 8 weeks has been shown to markedly reduce disease activity indices, fecal calprotectin levels, and serum CRP concentrations, while concurrently boosting albumin levels. This underscores the potential of vitamin D supplementation in mitigating intestinal inflammation in active colitis patients [111]. Notably, within the dosage range of 2000 IU to 50,000 IU per week, the therapeutic efficacy of vitamin D supplementation appears to be positively correlated with dosage escalation, with no reported serious adverse reactions. However, the precise optimal supplementation dosage remains to be definitively established [91,109]. Ongoing research aims to elucidate the most efficacious dosing regimen for achieving optimal therapeutic outcomes in IBD patients.

### 4.2. Implications of Vitamin D in Irritable Bowel Syndrome (IBS)

IBS represents a multifactorial chronic disorder characterized by recurrent symptoms of intestinal dysfunction, spasms, abdominal pain, and alterations in defecation patterns. [112]. Its etiology remains elusive, attributed to a complex interplay of environmental, genetic, and immune factors. Categorized by defecation habits, IBS encompasses diarrhea-predominant (IBS-D), constipation-predominant (IBS-C), and mixed-type (IBS-M) presentations [113]. Stress, depression, dysregulation of the brain–gut axis, dysbiosis of the gut microbiome, and intestinal infections are among the implicated factors contributing to IBS pathogenesis [114,115,116,117,118].

A considerable proportion of individuals with IBS may exhibit vitamin D deficiency, with serum vitamin D levels inversely correlating with disease severity. This underscores the potential therapeutic utility of vitamin D in IBS management [119,120]. In a randomized clinical trial involving IBS-D patients with vitamin D deficiency, supplementation with 50,000 IU of vitamin D over 9 weeks yielded improvements in IBS symptoms and reduction in serum interleukin-6 (IL-6) concentrations [121]. IL-6, a pro-inflammatory cytokine, plays a pivotal role in acute inflammatory responses and exerts regulatory effects on nerve function, regeneration, and metabolism. Elevated serum IL-6 levels have been documented in IBS patients [122,123]. However, it is noteworthy that achieving a therapeutic effect with vitamin D supplementation may necessitate doses exceeding the established safe upper limit. Notably, within safe dosage thresholds, daily supplementation with 3000 IU of vitamin D over 12 weeks failed to yield therapeutic benefits in IBS management [124]. Thus, optimization of vitamin D supplementation strategies for IBS treatment warrants further investigation to delineate effective dosing regimens and maximize therapeutic efficacy while ensuring safety. Continued research efforts are imperative to refine our understanding and approach to utilizing vitamin D as a therapeutic modality for IBS.

### 4.3. Influence of Vitamin D in Celiac Disease (CeD)

CeD stands as a chronic immune-mediated disorder of the small intestine, characterized by innate and adaptive immune responses triggered by dietary gluten ingestion in genetically predisposed individuals [125,126]. CeD manifests with diarrhea and mucosal damage, potentially leading to malnutrition, weight loss, growth retardation in children, and complications such as osteoporosis and intestinal lymphoma [127,128,129]. In recent years, the prevalence rate of CeD has been gradually increasing [130,131]. Currently, the primary treatment for CeD entails adherence to a strict gluten-free diet (GFD), which facilitates mucosal healing. However, GFD adherence may pose challenges in achieving adequate nutrition and necessitates careful dietary management in alignment with evidence-based guidelines. Studies have found that GFD can improve the composition of the gut microbiota but does not appear to safely restore the gut microbiota abundance [132]. Moreover, emerging non-dietary treatment modalities are under investigation [133,134,135,136].

Studies have suggested a correlation between the prevalence of CeD and birth season, implicating a potential association with ultraviolet B exposure and vitamin D status [137,138]. The intestinal mucosal permeability increased in patients with CeD. Gluten protein is converted into immunogenic molecules under the action of transglutaminase (TTG), which is presented to immune cells in the lamina propria to induce the production of inflammatory cytokines, resulting in intestinal villus atrophy and damage to intestinal barrier function [139,140]. Vitamin D supplementation has shown promise in ameliorating TJ barrier damage in CeD murine models and bolstering intestinal mucosal immunity [27,88,90]. Clinical studies have demonstrated that daily supplementation with 400 IU of vitamin D and 400 mg of calcium carbonate over 6 months in pediatric CeD patients on a GFD regimen effectively alleviates intestinal and systemic symptoms [141]. Furthermore, vitamin D and calcium supplementation exert notable therapeutic benefits in mitigating CeD-induced bone metabolic disorders in children [142]. These findings underscore the potential utility of vitamin D supplementation as an adjunctive therapeutic approach in CeD management, particularly in conjunction with GFD adherence and comprehensive nutritional support.

## 5. Limitations and Future Aspects

In the current landscape of vitamin D and gut health research, while numerous advancements have been made, several limitations persist. Foremost, the heterogeneity in experimental designs, sample sizes, and dosage of vitamin D across various studies poses challenges in drawing generalized and definitive conclusions. This diversity in methodologies often introduces inconsistencies and potential biases in the interpretation of results. Further research is warranted in the future to elucidate the impact of vitamin D on enhancing intestinal health across various physiological stages or in different intestinal pathological conditions, as well as determining the optimal dosage for supplementation. Furthermore, the intricate mechanisms underlying the role of vitamin D in modulating gut health, particularly its interactions with the gut microbiome and intestinal barrier function, remain incompletely elucidated. While we have gained valuable insights, a more comprehensive understanding of these mechanisms is crucial for developing targeted therapeutic strategies.

The understanding of the role of vitamin D in gut health has made significant progress; however, there still exist ample opportunities for further research and development. Looking forward, the integration of cutting-edge technologies such as metagenomics, single-cell transcriptomics, spatial transcriptomics, and metabolomics holds great potential for further elucidating the intricate role of vitamin D in gut health. These techniques can provide us with a deeper understanding of the molecular mechanisms and signaling pathways involved. In particular, future research should focus on investigating the specific role of vitamin D in intestinal diseases, such as inflammatory bowel disease and irritable bowel syndrome. Moreover, the utilization of big data and machine learning techniques can significantly enhance our ability to analyze vast amounts of research data. These techniques can reveal new associations, identify potential targets, and facilitate the development of precision medicine approaches tailored to individual patients.

## 6. Conclusions

The intestinal mucosal barrier serves as a critical line of defense, preventing the invasion of harmful substances and maintaining internal environmental stability. Vitamin D plays a multifaceted role in regulating the integrity of this barrier. It influences the structure and diversity of the gut microbiome, modulates the proliferation and differentiation of intestinal immune cells, affects the tight junctions between intestinal epithelial cells, and regulates immune factors crucial for mucosal immunity. Insufficient levels of vitamin D can disrupt the delicate balance of the gut microbiome, impair the intestinal mucosal barrier, and predispose individuals to intestinal diseases.

Despite significant advancements in understanding the impact of vitamin D on gut health, several fundamental questions remain unanswered and warrant further research attention. The effects of vitamin D on the gut microbiome are nuanced and varied, necessitating comprehensive exploration into the underlying mechanisms and optimal dosage thresholds. Additionally, elucidating the pathogenesis of intestinal diseases and unraveling the intricate mechanisms governing microbiota responses are areas of ongoing investigation.

A comprehensive examination of the interaction between vitamin D and the gut microbiome, intestinal epithelium, and immune responses holds immense potential for the development of rational strategies aimed at protecting or restoring the intestinal barrier. By delving deeper into these interactions, researchers can formulate evidence-based approaches to mitigate intestinal diseases and identify novel therapeutic targets for enhancing intestinal health. Thus, further insights into this dynamic interplay are essential for advancing our understanding and management of intestinal disorders.

## Figures and Tables

**Table 1:** Effects of vitamin D supplementation on the composition of gut microbiome in diverse populations.

Number of Participants	Country	Subject Information	Vitamin D Supplementation	Changes of Gut Microbiome	References
835	Australia	Australians aged 60–84 years	60,000 IU of vitamin D_3_/month for 5 years	No significant changes	[43]
36	Ireland	Healthy volunteers	60 µg vitamin D_3_ per day for four weeks	Bacteroidetes↓; Actinobacteria↑; *Bifidobacterium longum*↑; *Coprococcus*↑	[47]
26	Australia	18–60 years, with vitamin D deficiency (serum 25-hydroxyvitamin D (25(OH)D) concentration ≤ 50 nmol/L)	4000 IU/day for 16 weeks	*Lachnospira*↑; *Blautia*↓	[44]
18	United States	Adults ≥ 18 years with vitamin D deficiency (25-hydroxyvitamin D [25(OH)D] < 20 ng/mL)	Vitamin D_3_ (60 µg [2400 IU]/day) or 25-hydroxyvitamin D_3_ (20 µg/day) for 8 weeks	Firmicutes↓	[48]
74	Italy	35–75 years old colorectal cancer patients with resected colorectal cancer stage I–III in the last 24 months	2000 IU vitamin D_3_ per day for 1 year	*Leuconostoc pseudomesenteroides*↑; *Bacteroides gallinarum*↑; *Christensenella timonensis*↑; *Ruminococcus YE78*↑; *Faecalibacterium prausnitzii*↑; *Holdemanella biformis*↑; *Eubacterium brachy*↓; *Bacteroides coprocola*↓	[49]
870	United States	10–18 weeks of gestation	4400 IU vitamin D_3_ per day between enrollment and delivery	*Desulfovibrio*↓	[50]
18	Korea	Patients with *Clostridioides* difficile infections (CDI) and vitamin D insufficiency (vitamin D level < 17 ng/mL)	200,000 IU vitamin D_3_ per day for 2 weeks	Proteobacteria↓; Lachnospiraceae↑; Ruminococcaceae↑; Akkermansiaceae↑; Bifidobacteriaceae↑	[51]

Note: “↑” and “↓” represent an increase and a decrease in the abundance of the microbial community, respectively.

## Data Availability

Not applicable.
